# Deficient Complement Opsonization Impairs Mycobacterium avium Killing by Neutrophils in Cystic Fibrosis

**DOI:** 10.1128/spectrum.03279-22

**Published:** 2023-01-18

**Authors:** Patricia M. Lenhart-Pendergrass, Kenneth C. Malcolm, Emily Wheeler, Noel M. Rysavy, Katie Poch, Silvia Caceres, Kara M. Calhoun, Stacey L. Martiniano, Jerry A. Nick

**Affiliations:** a Department of Pediatrics, University of California San Diego, San Diego, California, USA; b Department of Medicine, University of Colorado School of Medicine, Aurora, Colorado, USA; c Department of Medicine, National Jewish Health, Denver, Colorado, USA; d Department of Pediatrics, University of Colorado School of Medicine, Aurora, Colorado, USA; Texas A&M University

**Keywords:** *Mycobacterium avium*, nontuberculous mycobacteria, neutrophils, cystic fibrosis, opsonization, complement C3, immunoglobulin M

## Abstract

Nontuberculous mycobacteria (NTM), including Mycobacterium avium, are clinically important pathogens in cystic fibrosis (CF). The innate immune response to M. avium remains incompletely understood. We evaluated the role of complement opsonization in neutrophil-mediated killing of M. avium. Killing assays were performed using neutrophils from healthy donors (HDs) and persons with CF (pwCF). Clinical isolates of M. avium were opsonized with plasma from HDs or pwCF, which was intact or heat-treated to inactivate complement. HD neutrophils had killing activity against M. avium opsonized with intact HD plasma and killing was significantly reduced when M. avium was opsonized with heat-inactivated HD plasma. When opsonized with HD plasma, CF neutrophils had killing activity against M. avium that was not different than HD neutrophils. When opsonized with intact plasma from pwCF, HD neutrophil killing of M. avium was significantly reduced. Opsonization of M. avium with C3-depleted serum or IgM-depleted plasma resulted in significantly reduced killing. Plasma C3 levels were elevated in pwCF with NTM infection compared to pwCF without NTM infection. These studies demonstrate that human neutrophils efficiently kill M. avium when opsonized in the presence of plasma factors from HD that include C3 and IgM. Killing efficiency is significantly lower when the bacteria are opsonized with plasma from pwCF. This indicates a novel role for opsonization in neutrophil killing of M. avium and a deficiency in complement opsonization as a mechanism of impaired M. avium killing in CF.

**IMPORTANCE** Mycobacterium avium is a member of a group of bacterial species termed nontuberculous mycobacteria (NTM) that cause lung disease in certain populations, including persons with cystic fibrosis (CF). NTM infections are challenging to diagnose and can be even more difficult to treat. This study investigated how the immune system responds to M. avium infection in CF. We found that neutrophils, the most abundant immune cell in the lungs in CF, can effectively kill M. avium in individuals both with and without CF. Another component of the immune response called the complement system is also required for this process. Levels of complement proteins are altered in persons with CF who have a history of NTM compared to those without a history of NTM infection. These results add to our understanding of how the immune system responds to M. avium, which can help pave the way toward better diagnostic and treatment strategies.

## INTRODUCTION

Nontuberculous mycobacteria (NTMs) include over 200 environmental species. A number of NTMs are pathogenic in susceptible hosts, including those with immunodeficiency or underlying lung disease such as cystic fibrosis (CF) (1). The Mycobacterium avium complex (MAC) is a group of slow-growing NTM comprised of several species (most commonly M. avium, Mycobacterium intracellulare, and Mycobacterium chimaera), which together account for approximately 70% of NTM-positive respiratory cultures in persons with CF (pwCF) in the United States (2). NTM infections are common in CF, and prevalence increases with age, ranging from 5 to 10% in the first decade of life up to a 5-year prevalence of 20% in the adult CF population (3–6). Guidelines exist for the diagnosis and treatment of NTM pulmonary disease (1, 7), with recommended treatment of a multidrug regimen administered for a minimum of a year, with numerous potential adverse effects and drug-drug interactions. Despite these burdensome treatment regimens, relapse and recurrent infection remain common. A central question is why a subpopulation of pwCF become infected with NTM, while the majority do not, despite similar risk factors, exposures, and disease severity. Improved understanding of the complex pathophysiology of NTM infection is critical to identifying host risk factors for infection and treatment failure (8).

Chronic neutrophilic inflammation is the hallmark of CF lung disease. High concentrations of proinflammatory cytokines promote neutrophil trafficking to the CF airway (9), and once there, CF neutrophils function abnormally (10). While neutrophils play a key role in host defense, the cycle of infection and persistent neutrophilic inflammation ultimately contributes to progression of lung disease. Neutrophils have been studied extensively in CF; however, their role against NTM infection in CF has been largely overlooked. Previous studies have investigated various aspects of neutrophil-mycobacterial interactions, including the role of phagocytosis, lysosomal killing, neutrophil extracellular traps (NETs), and neutrophil reactive oxygen species (ROS) with inconsistent findings, indicating a need for additional research (11). Relatively few studies have focused on M. avium, and the existing literature does not address whether there are differences in the context of CF. Our previous studies have investigated mechanisms of the neutrophil response to Mycobacterium abscessus, demonstrating that neutrophil killing of M. abscessus is dependent upon phagocytosis and NETs (12, 13). Limited data are available about mechanisms of the neutrophil response to M. avium, although it has been shown that opsonized M. avium is rapidly taken up and killed by neutrophils (14).

Opsonization, a process in which circulating plasma factors termed opsonins bind to pathogens, promotes pathogen recognition by innate immune cells and is a key driver of efficient phagocytosis and intracellular killing. Opsonization can be mediated by antibodies or by complement components. There are several known mechanisms by which CF bacterial pathogens, such as Pseudomonas aeruginosa and Staphylococcus aureus, can evade opsonization (15, 16). Immunoglobulins and complement factors are found not only in plasma but also in CF sputum, suggesting that opsonization can occur in the airways (17, 18). Despite this, pathogen opsonization can be disrupted by factors in the CF airway that exhibit an inhibitory effect, rendering opsonization less effective (19–23). To our knowledge, studies of opsonization in host defense against NTM infection in CF have not been reported. We hypothesized that in the subpopulation of pwCF who are infected with NTM, ineffective opsonization is one mechanism that allows M. avium to survive in the neutrophil-rich environment of the CF airway.

## RESULTS

### Heathy donor neutrophils effectively kill M. avium.

**FIG 1:**
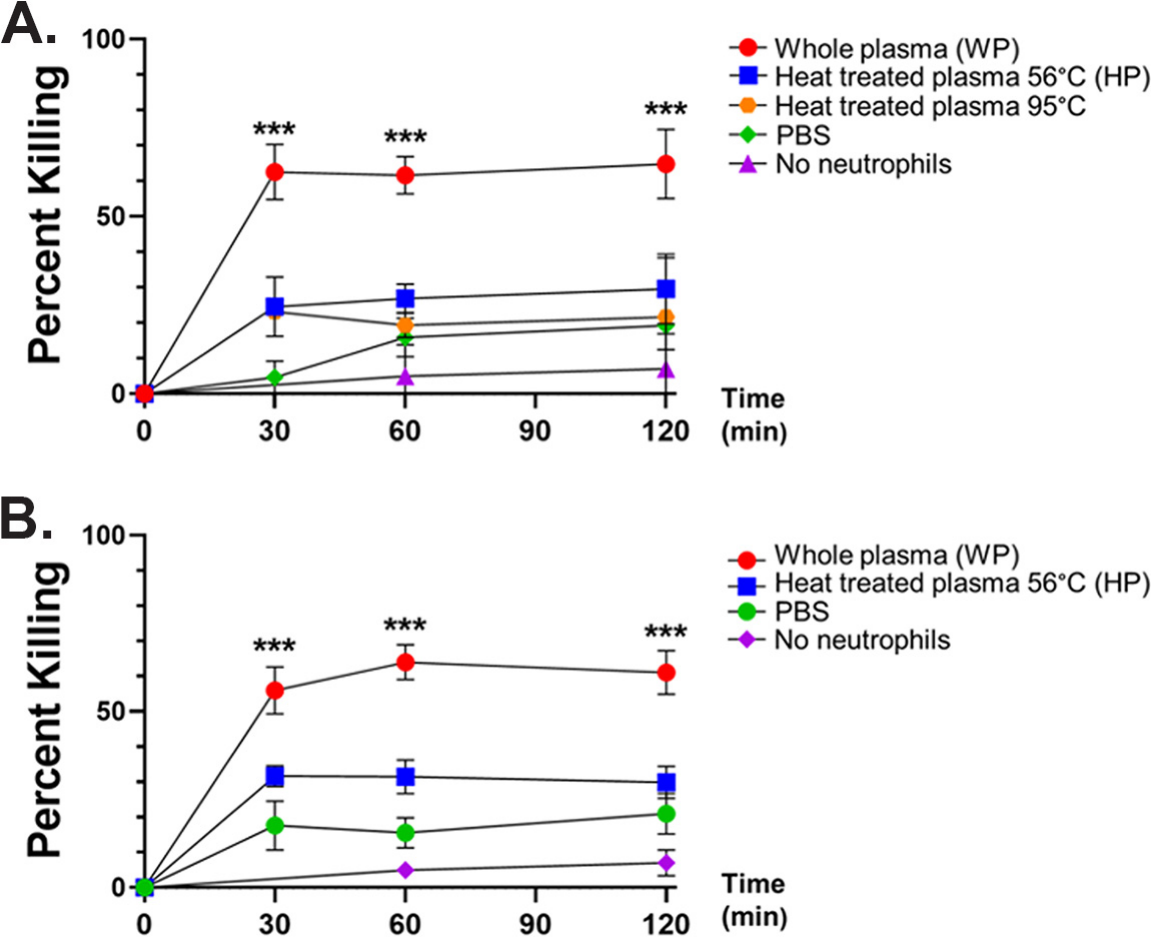
Healthy donor and cystic fibrosis (CF) donor neutrophil killing of M. avium. (A) Healthy donor neutrophils were incubated with M. avium opsonized with whole plasma (WP, red circles), plasma heat-treated at 56°C (HP, blue squares), or 95°C heat-treated plasma (orange hexagons) and in the absence of plasma components (phosphate-buffered saline [PBS], green diamonds) for a time course of up to 120 min. M. avium opsonized with WP and HP was also incubated for the same course without neutrophils (purple diamonds). Surviving M. avium colonies were compared to the corresponding *t* = 0 inoculum to calculate percentage of killing (*n* = 30 neutrophil donors). Killing assays were performed using M. avium isolate CF0002; however, identical killing assays were performed with two other M. avium clinical isolates from separate genetic lineages with consistent results. ***, *P* < 0.0001 by paired *t* test. (B) CF donor neutrophils were incubated with M. avium opsonized with WP (red circles) or HP (blue squares) or in the absence of plasma components (PBS, green circles) for a time course of up to 120 min. M. avium opsonized with WP and HP was also incubated for the same course without neutrophils (purple diamonds). Surviving M. avium colonies were compared to the corresponding *t* = 0 inoculum to calculate percentage of killing (*n* = 24 CF neutrophil donors). ***, *P* < 0.0001 by paired *t* test.

Neutrophils isolated from healthy donors were combined with M. avium for intervals of 0 to 120 min. Opsonization of M. avium with whole platelet-poor plasma (WP) pooled from healthy donors, which contains both complement and antibody opsonins, showed rapid and efficient killing of M. avium, with 61.6 ± 5.2% killed at 60 min. The percentage of killing decreased significantly to 26.9 ± 4.0% at 60 min (*P* < 0.0001 compared to WP conditions) when M. avium was opsonized with plasma heat treated at 56°C (HP), which inactivates complement. When M. avium was pretreated with phosphate-buffered saline (PBS) or plasma heat treated at 95°C, killing activity was also low (15.9 ± 5.5% and 19.3 ± 4.4%, respectively) and not significantly different than in HP conditions. There was also minimal killing activity when M. avium was opsonized with HP or WP and incubated without neutrophils (Fig. 1A).

### CF neutrophils effectively kill M. avium.

The killing capacity of neutrophils isolated from pwCF (*n* = 24) was tested in parallel with the healthy donor neutrophils. M. avium opsonized with healthy donor WP were killed rapidly and efficiently by CF neutrophils, not different than healthy donor neutrophils (63.9 ± 5.0% killing at 60 min, *P* = 0.53 compared to healthy donor neutrophil killing at 60 min). CF neutrophils also were significantly less able to kill M. avium opsonized with healthy donor HP (31.5 ± 4.8% at 60 min, *P* = 0.002 compared to WP conditions). When M. avium was pretreated with PBS rather than plasma, there was minimal killing activity by CF neutrophils (Fig. 1B).

To determine whether specific clinical characteristics modulated neutrophil killing in pwCF, we analyzed several variables often linked to severity of disease (Table 1). Comparison of M. avium percentage of killing at 60 min by CF neutrophils based on demographic or clinical characteristics (sex, use of cystic fibrosis transmembrane conductance regulator [CFTR] modulator, CFTR genotype, NTM infection history, current use of azithromycin, or current hospitalization for a pulmonary exacerbation) did not reveal any notable differences.

**TABLE 1:** Characteristics of CF Neutrophil Donors and M. avium killing^*a*^

Characteristic	HP		WP	
	Percent killing	*P* value	Percent killing	*P* value
Sex				
Male [*n* = 7]	43.0	0.99	74.1	0.97
Female [*n* = 17]	29.6		61.8	
CFTR modulator (current use)				
No HEMT [*n* = 8]	26.9	>0.99	59.6	0.99
+ HEMT [*n* = 16]	34.3		66.4	
CF genotype				
F508del/F508del [*n* = 12]	29.3	0.99	56.0	0.82
F508del/min [*n* = 12]	26.7		64.6	
Clinical status				
Outpatient [*n* = 7]	42.0	0.65	56.6	0.89
Inpatient [*n* = 17]	27.3		64.0	
NTM infection history				
− NTM [*n* = 15]	23.1	0.65	52.6	0.13
+ NTM [*n* = 9]	34.5		73.9	
Azithromycin (current use)				
− AZM [*n* = 10]	30.9	0.72	59.4	0.96
+ AZM [*n* = 14]	20.1		64.5	

a CF neutrophil killing of M. avium opsonized with HP or WP at *t* = 60 min did not significantly vary based on demographic and clinical characteristics of the CF neutrophil donor. The values were analyzed via one-way analysis of variance. AZM, azithromycin; CF, cystic fibrosis; CFTR, cystic fibrosis transmembrane conductance regulator; HEMT, highly effective modulator therapy; HP, heat-inactivated platelet-poor plasma; NTM, nontuberculous mycobacteria; WP, whole platelet-poor plasma.

### Mechanisms of M. avium killing by neutrophils.

**FIG 2:**
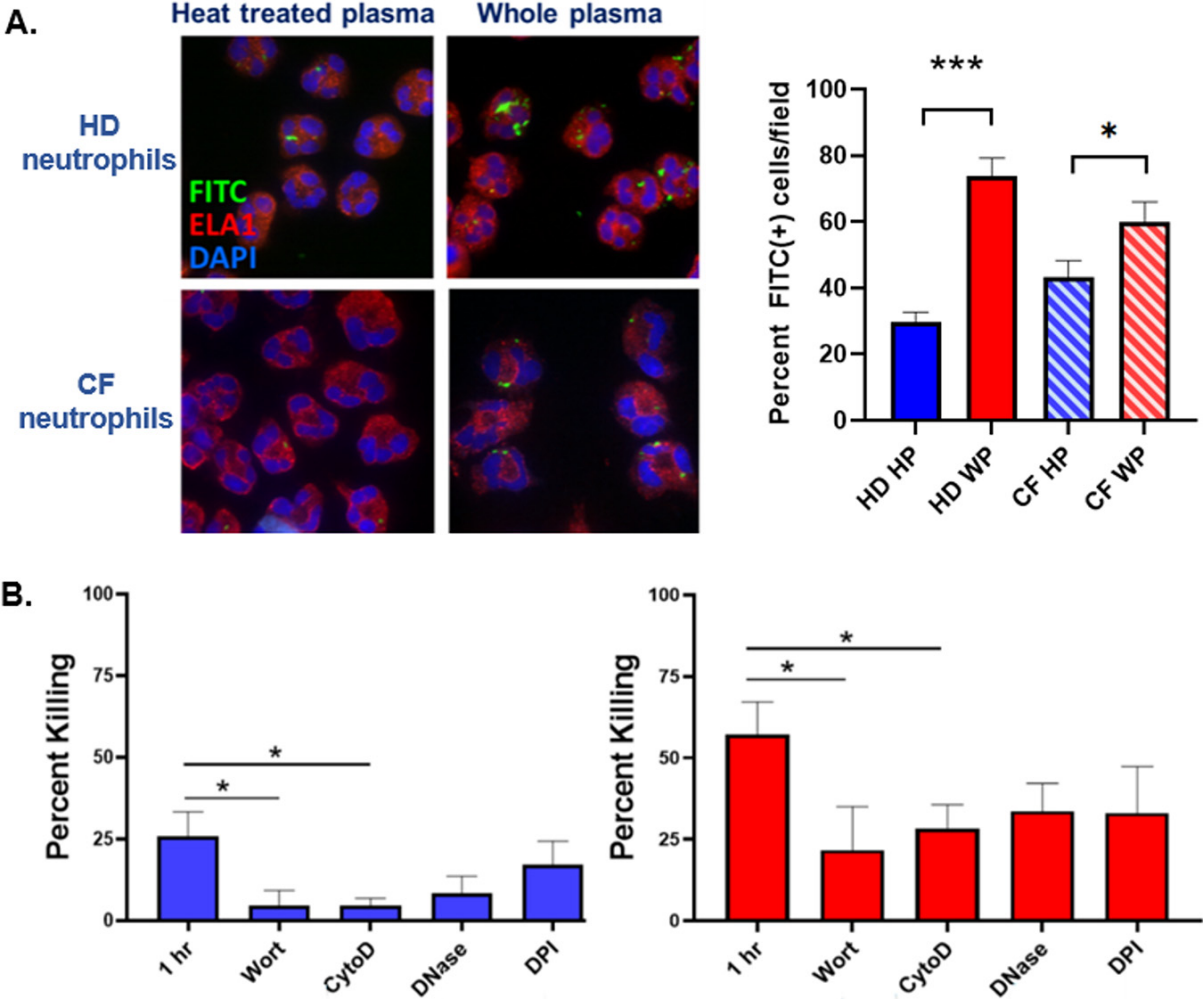
Mechanism of M. avium killing by neutrophils. (A) Healthy donor (HD) and CF neutrophils were combined with fluorescein isothiocyanate (FITC)-M. avium opsonized with HP or WP for 60 min, and confocal microscopy was performed for ELA1 (neutrophil elastase) and FITC. Images were quantified by calculating percent FITC(+) cells per 40× field from 3 to 4 fields of view from *n* = 3 separate experiments per condition. ***, *P* < 0.001; *, *P* = 0.04 by paired *t* test. (B) Healthy donor neutrophils were pretreated with wortmannin (Wort), cytochalasin D (CytoD), DNase, or diphenyleneiodonium chloride (DPI) and then incubated with M. avium opsonized with HP (blue) or WP (red) for 60 min. Surviving M. avium colonies were compared to the corresponding *t* = 0 inoculum to calculate percentage of killing (*n* = 5 to 8 neutrophil donors). *, *P* < 0.04 by paired *t* test.

Confocal microscopy was used to evaluate association of M. avium with neutrophils. Fluorescein isothiocyanate-labeled M. avium (FITC-Mav) opsonized with WP or HP was combined with healthy donor or CF donor neutrophils for 60 min. For both healthy and CF neutrophils, there was increased association between neutrophils and M. avium when the mycobacteria were opsonized with WP compared to HP (Fig. 2A), demonstrating that complement opsonization is necessary for efficient neutrophil uptake of M. avium.

Killing assays were also performed in the presence of well characterized inhibitors of phagocytosis (wortmannin or cytochalasin D), NETs (DNase), or ROS (diphenyleneiodonium chloride [DPI]), in order to evaluate the importance of various neutrophil killing pathways in response to M. avium. Inhibition of phagocytosis with wortmannin and cytochalasin D resulted in a significant decrease in the percentage of killing by healthy donor neutrophils in both WP and HP conditions, indicating that intracellular mechanisms are important in neutrophil killing of M. avium. Treatment with DNase or DPI had a weaker effect on killing that did not reach statistical significance (Fig. 2B).

### CF plasma is insufficient to allow for M. avium killing by healthy donor neutrophils.

**FIG 3:**
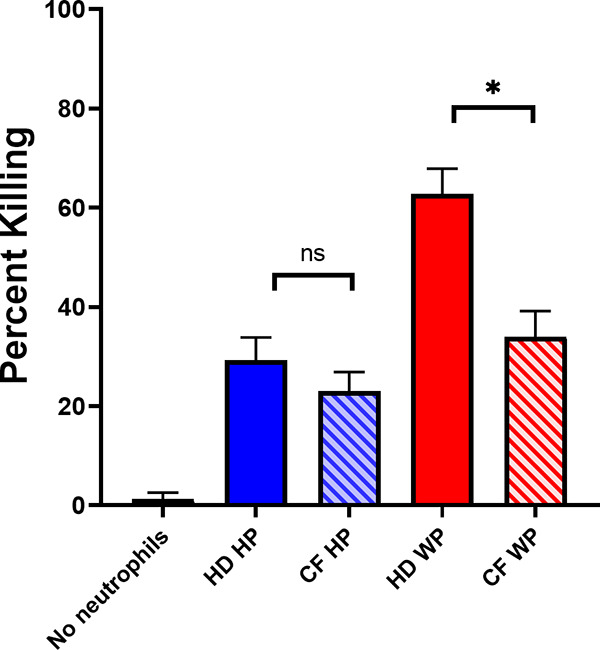
CF plasma opsonization and healthy donor neutrophil killing of M. avium. Healthy donor neutrophils were incubated with M. avium opsonized with HP from healthy donors (HD HP), HP from CF donors (CF HP), WP from healthy donors (HD WP), or WP from CF donors (CF WP) for 60 min. Surviving M. avium colonies were compared to the corresponding *t* = 0 inoculum to calculate percentage of killing (*n* = 8 to 13 for CF plasma; *n* = 30 for HD plasma). *, *P* = 0.01 by paired *t* test. ns, not significant.

Opsonization with CF plasma was then evaluated and compared to healthy donor plasma to determine whether CF affects the plasma-mediated killing of M. avium by neutrophils. Killing assays were performed using healthy donor neutrophils and M. avium opsonized with CF WP and CF HP. The percentage of killing at 60 min was determined. In HP conditions, there was no difference in neutrophil killing between CF HP and healthy donor HP. However, opsonization with CF WP resulted in a significantly lower percentage of killing compared to healthy donor WP (34.0 ± 5.2% compared to 62.7 ± 5.1%, *P* = 0.01) (Fig. 3), indicating impaired complement opsonization and killing by CF plasma.

### Complement C3 is required for neutrophils to effectively kill M. avium.

**FIG 4:**
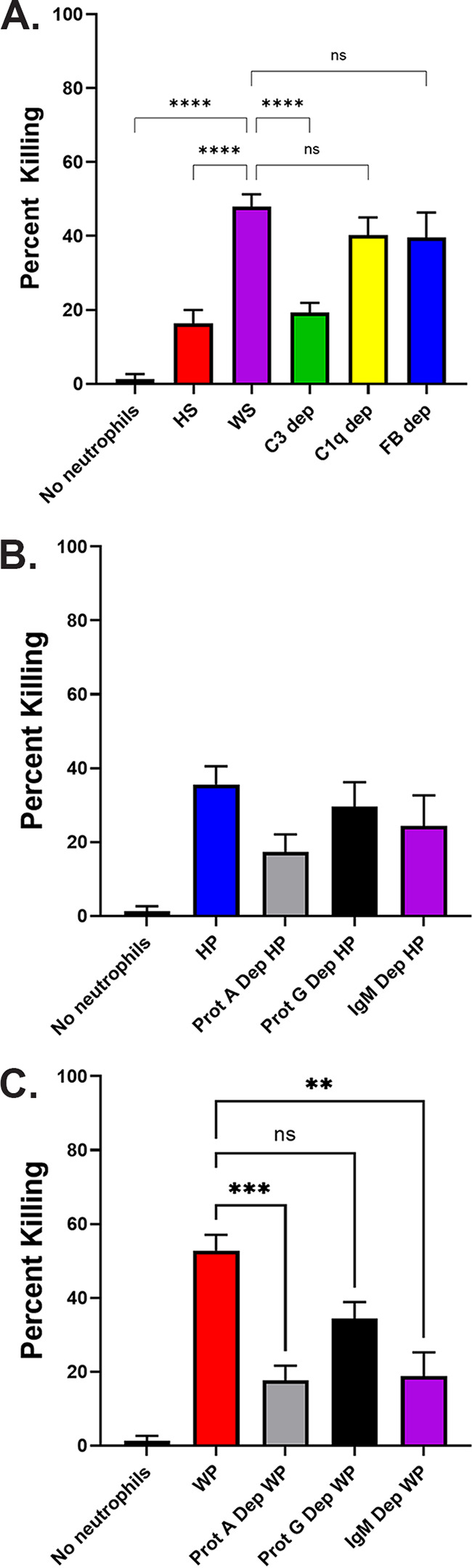
Complement and antibody depletion and neutrophil killing of M. avium. (A) Healthy donor neutrophils were incubated with M. avium opsonized with healthy donor heat-inactivated serum (HS), intact healthy donor serum (WS), or WS depleted (dep) of complement C3, C1q, or factor B (FB) for 60 min. Surviving M. avium colonies were compared to the corresponding *t* = 0 inoculum to calculate percentage of killing (*n* = 10 to 12). ****, *P* ≤ 0.0001 by one-way analysis of variance (ANOVA). (B) Healthy donor neutrophils were incubated with M. avium opsonized with healthy donor HP, HP treated with protein A- or protein G-agarose, and HP depleted of IgM for 60 min. Surviving M. avium colonies were compared to the corresponding *t* = 0 inoculum to calculate percentage of killing (*n* = 7 to 9). (C) Healthy donor neutrophils were incubated with M. avium opsonized with WP, WP treated with protein A- or protein G-agarose, and WP depleted of IgM for 60 min. Surviving M. avium colonies were compared to the corresponding *t* = 0 inoculum to calculate percentage of killing (*n* = 7 to 9). ***, *P* = 0.0003; **, *P* = 0.019 by one-way ANOVA.

Opsonization with plasma depleted of specific complement factors was then evaluated to determine which components are necessary for neutrophil killing of M. avium. Killing assays were performed using healthy donor neutrophils and M. avium opsonized with heat-inactivated healthy donor serum (HS), intact healthy donor serum (WS), and commercially available intact serum depleted of complement C3, C1q, and factor B (FB). Neutrophil killing of M. avium opsonized with serum versus plasma revealed no significant differences for both the intact and the heat-treated conditions. Complement depletion was confirmed via Western blot (Fig. S1A). Opsonization with C3-depleted serum resulted in significantly lower M. avium killing compared to WS at 60 min (19.3 ± 2.6% versus 47.9 ± 3.4%, *P* ≤ 0.0001). Killing with C3-depleted serum opsonization was very similar to killing with HS opsonization, indicating that complement depletion can account for the neutrophil killing defect observed with HS. Depletion of C1q and factor B did not significantly affect M. avium killing efficiency (Fig. 4A).

### Immunoglobulin M is required for neutrophils to effectively kill M. avium.

Opsonization with plasma depleted of antibody components was then evaluated to determine which antibody classes are necessary for neutrophil killing of M. avium. Killing assays were performed using healthy donor neutrophils and M. avium opsonized with healthy donor HP or WP and protein A- or protein G-agarose-treated HP or WP. Antibody depletion was confirmed by Western blot (Fig. S1B). Protein A-agarose depletes IgG1, IgG2, IgG4, IgA, and IgM. Protein A treatment had no significant effect on killing in HP conditions (Fig. 4B); however, opsonization with protein A-depleted WP resulted in significantly lower M. avium killing efficiency compared to WP that was not depleted (17.7 ± 3.9% versus 52.8 ± 4.3%, *P* = 0.0013) (Fig. 4C). Protein G-agarose depletes IgG subclasses 1 to 4 but has minimal effect on IgA or IgM, and opsonization with protein G-depleted plasma had a weaker effect on neutrophil killing of M. avium in HP or WP conditions that did not reach significance (Fig. 4B and C).

The difference in the depleted immunoglobulins between protein A and protein G suggests that IgM is an important opsonin. Given the known role of IgM in promoting complement opsonization (24, 25), specific IgM depletion was also tested. M. avium was opsonized with healthy donor HP and WP that was depleted of IgM, and killing assays were performed with healthy donor neutrophils. IgM depletion was confirmed via Western blot (Fig. S1C). IgM depletion had no significant effect on killing in HP conditions (Fig. 4B); however, opsonization with IgM-depleted WP resulted in significantly lower M. avium killing compared to WP that was not depleted (18.8 ± 6.4% versus 52.8 ± 4.3%, *P* = 0.0019) (Fig. 4C), implicating IgM as an important antibody component in the neutrophil killing response to M. avium.

### Complement C3 and IgM directly opsonize M. avium.

**FIG 5:**
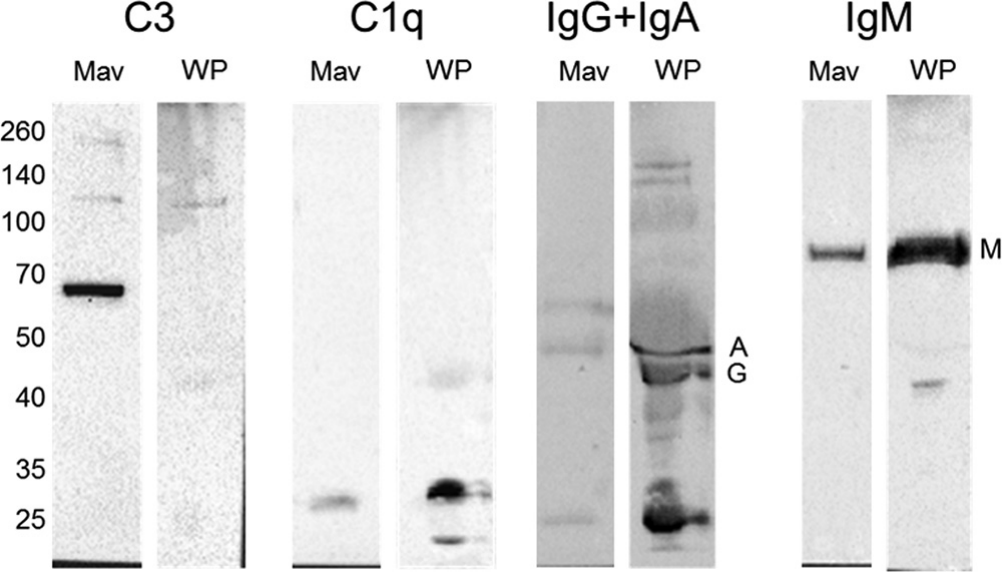
Complement and antibody opsonization of M. avium. Comparison of protein levels of C3, C1q, IgG, IgA, and IgM bound to M. avium (Mav) opsonized with WP compared to WP alone via Western blot. C3 and IgM (M) were both found to associate with M. avium opsonized with WP. C1q, IgG (G), and IgA (A) were also associated with opsonized M. avium in but in small amounts.

To confirm that C3 and IgM participate in M. avium opsonization, we performed Western blots evaluating protein levels of complement C3, C1q, IgG, IgA, and IgM bound to WP opsonized M. avium, compared to WP alone (Fig. 5). Robust levels of C3 and IgM protein were bound to WP opsonized M. avium, providing further evidence that these components act as opsonins. C1q, IgG, and IgA were also associated with M. avium but only in small amounts, suggesting that these factors are less important in M. avium opsonization.

### Complement levels in CF plasma by NTM infection status.

**FIG 6:**
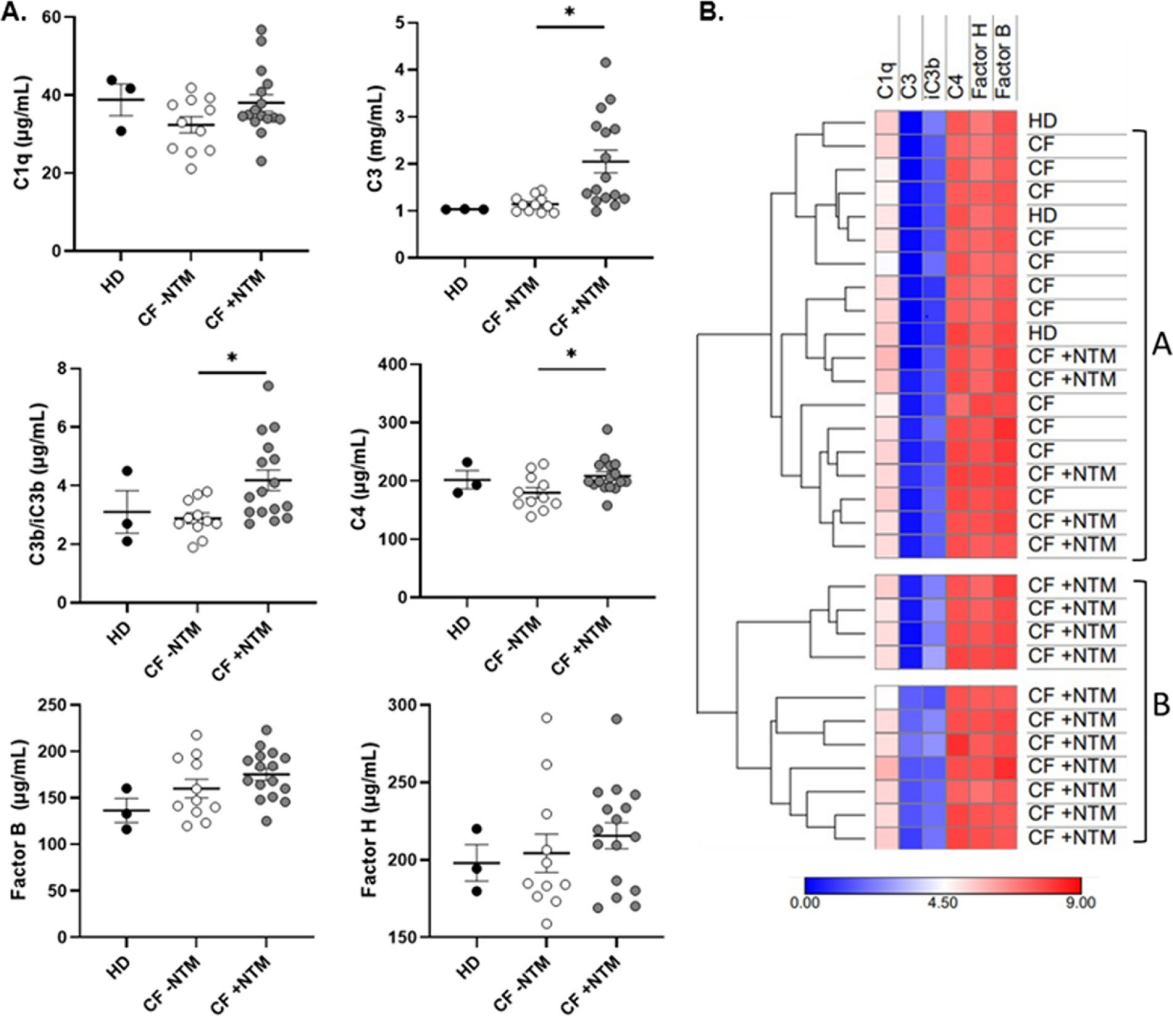
Complement levels in persons with CF (pwCF) with and without nontuberculous mycobacteria (NTM) infection. (A) Plasma levels of complement factors C1q, C3, C3b/iC3b, C4, factor B, and factor H in healthy donors, pwCF with no history of NTM infection (CF −NTM), and pwCF with a history of NTM infection (CF +NTM). Complement C3 and C3b/iC3b levels were significantly higher in pwCF with a history of NTM compared to pwCF without a history of NTM (*P* = 0.01 and *P* = 0.02, respectively, by one-way ANOVA) and were higher than in healthy donors. Complement C4 levels were also higher in pwCF with a history of NTM compared to those without (*P* = 0.045), but levels were similar to those of healthy donors. (B) Hierarchical clustering of individual subject complement profiles. Unsupervised clustering of complement profiles revealed two primary clusters representing low (cluster A) and high (cluster B) levels. All HD subjects and all CF without NTM fell within cluster A, along with 5 of 16 (31%) of CF +NTM subjects. Cluster B was comprised entirely of CF +NTM subjects (*P* = 0.0003 by two-tail Fisher’s exact test, for subjects with CF).

Levels of six complement components (C1q, C3, C3b/iC3b, C4, factor B, and factor H) in plasma specimens from healthy donors and pwCF with and without a known history of NTM infection were analyzed via Luminex (Fig. 6). Levels of complement C3 were significantly elevated in pwCF with NTM infection compared to pwCF without NTM infection (*P* = 0.01). Similarly, C3b/iC3b levels were significantly higher in pwCF with NTM compared to pwCF without NTM (*P* = 0.02). Complement C4 levels were also higher in pwCF with a history of NTM compared to those without (*P* = 0.045); however, C4 levels in pwCF with NTM were similar to healthy donors. The levels of complement factors C1q, factor B, and factor H were not significantly different between pwCF with and without NTM infection (Fig. 6A). Mean values for all tested complement factors are reported in Table S1. Unsupervised hierarchical clustering of individual subject complement profiles revealed two primary clusters comprised of low and high complement levels. All HD subjects and all CF without NTM were grouped in the low complement cluster, along with 5 of 16 (31%) of pwCF with a history of NTM. The high complement cluster was comprised entirely of pwCF with NTM (*P*= 0.0003), suggesting a distinct complement activation profile in some pwCF with NTM infection (Fig. 6B).

## DISCUSSION

NTM infections, of which M. avium is the most common in the United States (26), are a challenging clinical problem, particularly for pwCF. The innate immune response to M. avium has not been well characterized, and the role of opsonization in the neutrophil response to M. avium has been minimally addressed in the existing literature. Furthermore, although neutrophilic airway inflammation is characteristic of CF, little is known about how neutrophil interactions with M. avium may differ in CF. Given the ubiquitous presence of NTM species in the environment, most individuals are exposed to these potential pathogens on a frequent basis, yet only a subset develop infection or resultant pulmonary disease. CF is a clearly established risk factor for NTM pulmonary disease; pre-existing airway inflammation, chronic infection, mucous stasis, and structural lung disease are presumed to explain this increased risk, yet our mechanistic understanding of the innate immune response to NTM in CF remains lacking. In the present study, we addressed several of these gaps in our understanding of neutrophil interactions with M. avium, the role of opsonization in the neutrophil response, and how these host-pathogen interactions differ in some pwCF.

This study demonstrates that healthy donor neutrophils can efficiently kill M. avium. This finding is in agreement with the limited existing literature on the subject. Hartmann et al. found that opsonized M. avium is efficiently killed by human neutrophils (14), and prior work by our group has also demonstrated efficient killing of nonopsonized M. abscessus by healthy donor neutrophils (13). In addition to providing evidence in support of neutrophil killing of NTM, several mechanistic aspects of this killing response were evaluated. An important role for opsonization, particularly complement-dependent opsonization, was detected in healthy donor neutrophil killing of M. avium, which has not been previously characterized. Neutrophil killing of nonopsonized M. abscessus has been previously shown to depend upon both phagocytosis and extracellular killing mechanisms (13). Here, we provide evidence that healthy donor neutrophil killing of M. avium is also dependent upon phagocytosis but is not dependent upon extracellular mechanisms, including extracellular traps. Together, these findings describe a novel, important role for complement-dependent opsonophagocytosis in neutrophil killing of M. avium.

We found that both complement C3 and IgM are important in M. avium killing by healthy neutrophils. Complement C3 is a central component of all three of the complement pathways (classical, alternative, and lectin) and C3 fragments, including C3b, are important opsonins (27). Depletion of complement C3 significantly reduced M. avium killing by neutrophils. Depletion of other complement components, including C1q, a component of the classical pathway, and factor B, a component of the alternative pathway, did not have a robust effect on killing. Our finding that neutrophil killing of M. avium is dependent upon IgM and C3 but independent of C1q may indicate that the killing response is not occurring through canonical antibody-mediated activation of classical complement pathway via C1q. Instead, it is possible that noncanonical complement activation pathways are involved. Interactions between antibodies, including IgM and the lectin complement pathway, have been described; for example, some IgM glycoforms can bind to mannose-binding lectin, which activates the lectin pathway (28–30). Complement activation via IgM-ficolin interaction has also been reported (31). Finally, an additional possibility is that the classical complement pathway is involved in the neutrophil response to M. avium, but the activity of other complement activation pathways is upregulated in a compensatory fashion when C1q is inhibited. This can be addressed in future experiments in which multiple complement pathways are simultaneously inhibited. We also found that M. avium killing occurred at very low levels in the presence of plasma components when neutrophils were absent, indicating that the killing response detected in our studies was neutrophil-mediated and not a direct effect of terminal complement activation.

Interestingly, depletion of IgM using protein A (which also depletes IgA and some IgGs) or specific depletion of IgM alone resulted in impaired neutrophil-mediated M. avium killing, suggesting a major role of IgM in M. avium opsonization. Specific depletion of IgG with protein G did not significantly alter killing. Secreted IgM, when bound to a pathogen surface, can initiate complement activation, leading to opsonization and pathogen destruction (24, 25). IgM can be divided into two classes, natural IgM, which is produced even in the absence of pathogen exposure, and immune (or antigen-induced) IgM, which is produced following exposure to an antigen or a pathogen. Although historically IgM was thought to be part of a transient initial response to exposure, there is growing evidence that IgM may also be involved in long-lasting adaptive immune responses to pathogens (32). The role of natural versus immune IgM has not been well studied in regard to NTM infection, although antibody responses to NTM have been described both in general NTM pulmonary disease cohorts (33) and in pwCF with NTM infection (34). MAC infection has also been reported in individuals with selective IgM deficiency (35). Based on our findings, we propose IgM-mediated activation of complement opsonization as one mechanism of M. avium recognition and killing by human neutrophils. Future studies are needed to further define the role of IgM in M. avium infection and to evaluate the role of various complement cascade components in more detail.

The killing response of neutrophils from pwCF against M. avium was also evaluated. Deficient killing activity by CF neutrophils against typical bacterial pathogens, including P. aeruginosa and S. aureus, has been described (36–38). Interestingly, we showed that M. avium killing efficiency by CF neutrophils is very similar to the M. avium killing efficiency of healthy donor neutrophils (when M. avium is opsonized with healthy donor plasma). This result suggests that the neutrophil killing response against NTM is not CFTR dependent and that the increased susceptibility of CF hosts to NTM pulmonary disease may be caused by the altered airway milieu rather than defective intrinsic killing by CF neutrophils. Importantly, CF neutrophil killing did not vary based on demographic or clinical variables of the CF neutrophil donors, including current use of a CFTR modulator. Similar to our findings for healthy donor neutrophils, CF neutrophil killing of M. avium was also highly dependent upon complement opsonization. We also demonstrated that opsonization with CF plasma was not sufficient for efficient M. avium killing by healthy donor neutrophils, suggesting that opsonization may be deficient in CF.

From a translational perspective, we tested the levels of complement components in the plasma of pwCF with and without a known history of NTM infection. Various complement components have been tested in CF, in both blood and airway samples, with variable results. For example, several studies have demonstrated that the proinflammatory C5a effector is elevated in CF airway specimens compared to controls. Elevated C5a correlates with increased disease severity in CF, whereas the anti-inflammatory C3a correlated with increased lung function (39, 40). In studies done in the 1970s, C3 levels and activation were shown to be unchanged in a CF population compared to controls (41, 42). C4 deficiency has been associated with increased risk for MAC disease in a non-CF population (43). Our results show a robust increase in complement C3 levels in pwCF with NTM infection compared to pwCF without NTM. Similarly, levels of the downstream effectors of C3, C3b/iC3b, were significantly elevated in pwCF with NTM compared to those without. Complement C4 levels were also statistically higher in the NTM group, although this may not be of clinical significance given the small difference in values compared to healthy donors. While complement levels can be increased in response to inflammation and infection, our findings do not appear to be indicative of nonspecific inflammation in CF, given that the other complement components assessed were not significantly elevated. Additionally, C3 and C3b/iC3b were elevated only in pwCF with NTM, although both the NTM and non-NTM CF groups are likely to have increased inflammation relative to healthy controls. One explanation for this finding is that C3 elevation in pwCF with NTM infection occurs because C3 is a critical component of the neutrophil killing response and may control systemic infection. A limitation of these findings is that the complement assay measures levels but not function, which could vary. Sputum-independent diagnostics for NTM are a topic of great interest in the CF community, and future studies could evaluate C3 as a serum biomarker for NTM infection in pwCF. Heat map clustering analysis showed distinct complement activation profiles in pwCF with NTM, which could also have diagnostic utility.

One important area for future study is to correlate our findings to the CF airway. Numerous studies have also demonstrated the presence of complement and antibody components in the airway (17, 18), although these components are subject to cleavage by proteases in the CF airway that can render them ineffective (19–23, 44, 45). Using a proteomics approach, Hayes et al. recently demonstrated that complement components were largely absent from bronchoalveolar lavage fluids in pwCF both with and without NTM infection (46). In the context of our findings that complement is an important component of the neutrophil response to M. avium, this may indicate that local complement deficiency in the lung could be a particular susceptibility factor in NTM infection. Elevated plasma C3 in persons with CF with NTM infection, as demonstrated in our complement assays, may be effective for preventing disseminated NTM infection. C3 elevation could also occur as compensatory response to complement deficiency in the airway yet may not be sufficient for control of NTM infection in the airway environment. Future studies are needed to further evaluate innate immune interactions with NTM in airway specimens.

Taken together, our results provide an expanded understanding of neutrophil interactions with M. avium in healthy donors and provide new insights into how these interactions are similar and how they differ in cystic fibrosis. Improved understanding of the basic pathophysiology of these complex infections is essential to better understand host susceptibility factors and for future development of novel diagnostic and therapeutic approaches (47).

## MATERIALS AND METHODS

### Bacterial strains, media, and culture conditions.

M. avium isolates were obtained from the sputum of CF subjects with NTM pulmonary disease who were enrolled in the PRospective Evaluation of NTM Disease In CysTic Fibrosis study (PREDICT, NCT02073409). Identification of each isolate was confirmed by whole-genome sequencing, as previously described (48). M. avium isolate CF0002 was used for most experiments and was propagated from frozen aliquots in 7H9 broth supplemented with 0.5 g/liter bovine albumin fraction V, 0.2 g/liter dextrose, and 0.3 mg/liter catalase (ADC; BD Biosciences, Franklin Lakes, NJ) at 37°C with shaking at 300 rpm for 7 to 10 days. The cultures were sonicated using a Fisher Sonic Dismembrator 100 and then were adjusted to optical density at 600 nm (OD_600_) of 1.0 in phosphate-buffered saline (PBS), corresponding to approximately 1× 10^9^ CFU/mL. Most assays were completed using a single M. avium isolate (CF0002); two other M. avium isolates (CF0012 and CF0052) were also tested with very similar findings as CF0002, so results from all isolates were combined.

### Plasma preparation and opsonization.

These studies were approved by the National Jewish Health Institutional Review Board, and informed consent was obtained from all specimen donors. The study was conducted in accordance with the Declaration of Helsinki. Whole blood was collected in EDTA K5 tubes from healthy donors and pwCF, and plasma was obtained following manufacturer’s guidelines. Whole human platelet-poor plasma (WP) samples were combined and aliquoted into three to five donor pools. Heat-inactivated human platelet-poor plasma (HP) was prepared by incubating WP at 56°C for 30 min or at 95°C for 30 min as indicated. To prepare opsonized M. avium, 50 μL of WP or HP was added to 100 μL of M. avium at OD_600_ = 1.0. Complement C3-, C1q-, and factor B-depleted sera and control human serum (Complement Technology Inc, Tyler, TX) were also used to opsonize M. avium. Antibody-depleted plasma was prepared by incubating 300 μL of protein A- or protein G-agarose (Invitrogen, Waltham, MA) with 300 μL of WP at 37°C for 1 h with rotational mixing, followed by centrifuging at maximum speed and collecting the supernatant. IgM-depleted plasma was prepared by incubating 600 μL of anti-IgM-agarose (Sigma-Aldrich, St. Louis, MO) with 300 μL of WP and following the same procedure as the protein A and G plasma preparation. Antibody depletion was confirmed by Western blot.

### Neutrophil isolation.

Neutrophils were isolated from healthy donors by the plasma Percoll method as described previously (49, 50). For CF neutrophils, peripheral blood mononuclear cells were isolated by density centrifugation using Lymphoprep (Stemcell Technologies, Cambridge, MA) and removed from the gradient; red blood cell (RBC) lysis was then performed with H_2_O, and isolated neutrophils were used for downstream applications. Mononuclear cell contamination was minimal and equivalent between the two neutrophil preparation methods (≤2.5%).

### Killing assays.

Neutrophil killing assays were conducted as previously described (13). Briefly, 1× 10^6^ neutrophils were combined with M. avium at a multiplicity of infection of approximately 1.25:1. Inhibitors (wortmannin, cytochalasin D, DNase, or DPI), when used, were preincubated with neutrophils for 20 min prior to adding bacteria. The tubes were centrifuged at 2,000 × *g* for 1 min, and pelleted cells were incubated 37°C for 10 min, followed by resuspension and incubation for up to 2 h. Triton X-100 (0.1%) PBS was added, and samples were vortexed. The samples were serially diluted in saline and plated on 7H10 agar supplemented with OADC. The plates were incubated at 37°C for 7 to 10 days. Colonies were counted and compared to colony counts at the initiation of infection. *n* for all killing assays represents separate opsonization reactions and separate neutrophil donors.

### Phagocytosis of M. avium.

FITC-labeled M. avium (FITC-Mav) was prepared and bacterial localization by confocal microscopy was performed as previously described (50). FITC-Mav were sonicated, washed, and resuspended into 0.5 mL of PBS. FITC-Mav was combined with neutrophils for 1 h at a multiplicity of infection of approximately 5:1. The cells were cytocentrifuged to microscope slides and then air-dried. The slides were processed with rabbit anti-neutrophil elastase antibody (Abcam, Boston, MA), 4′,6-diamidino-2-phenylindole (DAPI), and Alexa Fluor 555-conjugated goat anti-rabbit (Invitrogen, Waltham, MA) and imaged using a Zeiss Axiovert 200M microscope. Images were quantified by calculating percent FITC(+) cells per field.

### Western blots.

M. avium was opsonized as described above, using three times the volumes of each component, for 30 min at 37°C. The cells were washed three times with PBS, resuspended in sample buffer, and treated for 5 min at 95°C. Proteins were separated on 10% SDS polyacrylamide gels and transferred to nitrocellulose membranes. The blots were probed with anti-C3 (BioLegend, 846302), anti-C1q (Novus NB100-64420), anti-IgA (Southern Biotech 2050-05), anti-IgG (Abcam ab97175), or anti-IgM (Southern Biotech 2020-05), as indicated, overnight. Anti-Ig antibodies were horseradish peroxidase (HRP)-conjugated and detected directly with ECL reagent, and C3 and C1q were incubated for 1 h with anti-rabbit (Cell Signaling 7074) or anti-sheep-HRP (Southern Biotech 6150-05). Detection was performed on a Bio-Rad ChemiDoc MP Imaging System and Image Lab software (version 5.2.1).

### Complement assay.

Plasma specimens were collected from healthy donors (*n* = 3) and pwCF with (*n* = 16) and without (*n* = 11) known NTM infection. The samples were analyzed using Human Complement Panel II (Millipore, Burlington, MA) in a Luminex Magpix. Analytes tested included C1q, C3, C3b/iC3b, C4, factor B, and factor H. Hierarchical clustering was performed using Morpheus analysis software (Broad Institute) with log_2_-normalized values, using Euclidean distance metric and complete linkage.

### Statistical analysis.

The data are presented as means ± standard error of the mean (SEM) and were analyzed by *t* test, one-way analysis of variance (ANOVA), or Fisher’s exact test as noted in figure legends. All analyses were performed using GraphPad Prism 9. Significance was set at a *P* value of 0.05.

### Data availability.

The data that support the findings of this study are openly available in Mendeley at https://doi.org/10.17632/yj6gdcx2nd.1.
