# Supplementary material for: Deficient Complement Opsonization Impairs Mycobacterium avium Killing by Neutrophils in Cystic Fibrosis
Source: Microbiol Spectr. 2023 Jan 18;11(1):e03279-22. doi: 10.1128/spectrum.03279-22 (PMC9927418; doi:10.1128/spectrum.03279-22)
Supplement: Supplemental file 1 — Supplemental material. Download spectrum.03279-22-s0001.pdf, PDF file, 0.3 MB [file spectrum.03279-22-s0001.pdf]

Supplemental Figure 1

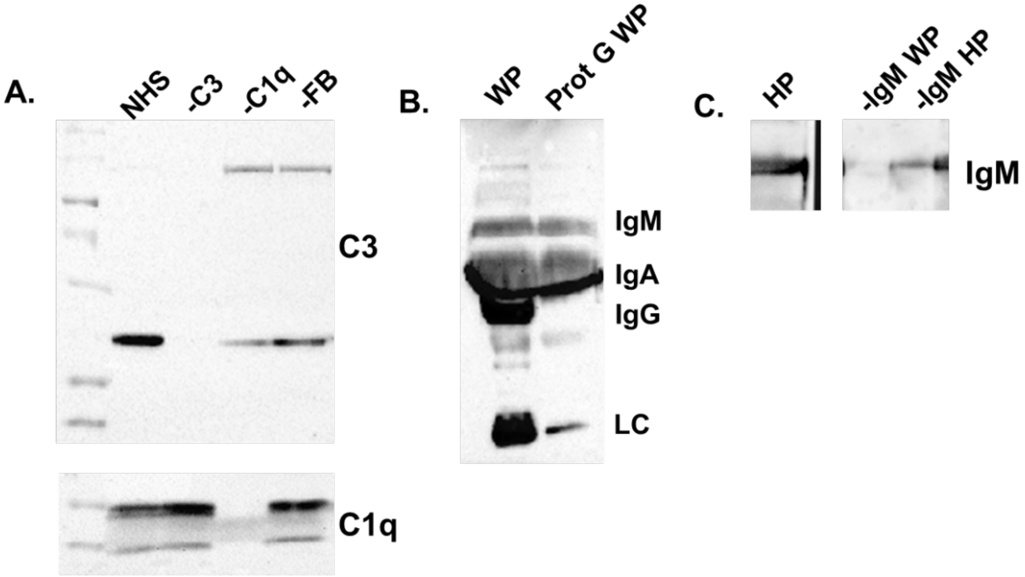

A. Protein levels of complement C3 and C1q were tested by western blotting in normal human serum (NHS), and C3 (-C3), C1q (-C1q), and Factor B (-FB) depleted sera. As expected, there was no detectable C3 in the C3 depleted serum, and no C1q detected in the C1q depleted sera. Factor B depletion was not tested.

B. Protein levels of IgM, IgA, IgG, and light chain (LC) were tested by western blotting in WP and Protein G treated WP. As expected, there was a decrease in IgG levels with Protein G treatment, with minimal effect on IgM or IgA. Protein A depletion was not tested.

C. Protein levels of IgM were tested by western blotting in HP and IgM (-IgM) depleted HP and WP. As expected, levels of IgM were reduced in the IgM depleted conditions.

Supplemental Table 1

	**HD**	**CF -NTM**	**CF +NTM**
**C1q** (μg/mL)	38.8	32.4	38.0
**C3** (mg/mL)	1.0	1.2	2.2
**C3b/iC3b** (μg/mL)	3.1	2.9	4.2
**C4** (μg/mL)	201.8	179.9	208.7
**Factor B** (μg/mL)	136.6	160.1	175.3
**Factor H** (μg/mL)	198.1	204.4	215.6

Supplemental Table 1. Mean complement protein levels in healthy donor (HD), pwCF without NTM (CF -NTM), and pwCF with NTM (CF +NTM).
